# Promoter Methylation of RASSF1A Associates to Adult Secondary Glioblastomas and Pediatric Glioblastomas

**DOI:** 10.5402/2012/576578

**Published:** 2012-01-09

**Authors:** Jorge Muñoz, María del Mar Inda, Paula Lázcoz, Idoya Zazpe, Xing Fan, Jorge Alfaro, Teresa Tuñón, Juan A. Rey, Javier S. Castresana

**Affiliations:** ^1^Unidad de Biología de Tumores Cerebrales, Universidad de Navarra, 31008 Pamplona, Spain; ^2^Gene Expression and Cancer Group, Vall d'Hebrón Instituto de Oncología, 08035 Barcelona, Spain; ^3^Departamento de Ciencias de la Salud, Universidad Pública de Navarra, 31008 Pamplona, Spain; ^4^Servicio de Neurocirugía, Hospital de Navarra, 31008 Pamplona, Spain; ^5^Department of Neurosurgery, University of Michigan Medical School, Ann Arbor, MI 48109, USA; ^6^Servicio de Anatomía Patológica, Hospital Miguel Servet, 50009 Zaragoza, Spain; ^7^Servicio de Anatomía Patológica, Hospital de Navarra, 31008 Pamplona, Spain; ^8^Unidad de Investigación IdiPAZ, Hospital Universitario La Paz, 28046 Madrid, Spain

## Abstract

While allelic losses and mutations of tumor suppressor genes implicated in the etiology of astrocytoma have been widely assessed, the role of epigenetics is still a matter of study. We analyzed the frequency of promoter hypermethylation by methylation-specific PCR (MSP) in five tumor suppressor genes (PTEN, MGMT, RASSF1A, p14^ARF^, and p16^INK4A^), in astrocytoma samples and cell lines. RASSF1A was the most frequently hypermethylated gene in all grades of astrocytoma samples, in cell lines, and in adult secondary GBM. It was followed by MGMT. PTEN showed a slight methylation signal in only one GBM and one pilocytic astrocytoma, and in two cell lines; while p14^ARF^ and p16^INK4A^ did not show any evidence of methylation in primary tumors or cell lines. In pediatric GBM, RASSF1A was again the most frequently altered gene, followed by MGMT; PTEN, p14 and p16 showed no alterations. Lack or reduced expression of RASSF1A in cell lines was correlated with the presence of methylation. RASSF1A promoter hypermethylation might be used as a diagnostic marker for secondary GBM and pediatric GBM. Promoter hypermethylation might not be an important inactivation mechanism in other genes like PTEN, p14^ARF^ and p16^INK4A^, in which other alterations (mutations, homozygous deletions) are prevalent.

## 1. Introduction

The role of epigenetics in the control of gene expression is having an increasing interest in cancer research. Aberrant cytosine methylation can promote tumor initiation or progression, both by excess (promoter hypermethylation of tumor suppressor genes) and defect (global genome hypomethylation, which can lead to chromosomal instability, reactivation of parasitic sequences, and loss of genomic imprinting).

In the last decade, several authors have reported the importance of epigenetic silencing of tumor suppressor genes in a wide range of human tumors. Some of these tumor suppressor genes inactivated by cytosine methylation on their promoters are p14^ARF^, APC, MLH1, and MGMT in colon cancer [1], DAPK in bladder superficial carcinoma [2], CDKN2B in leukemia and lymphoma [1], GSTP1 in prostate cancer and hepatocarcinoma [3], Caspase-8 in neuroblastoma [4], and RASSF1A in medulloblastoma, Wilms' tumor, and neuroblastoma [5]. Some of the main functions of these genes affect cell cycle control (CDKN2B, p14^ARF^), DNA damage repairing (MGMT, MLH1), cell adhesion (APC), apoptotic response (Caspase-8, DAPK), and microtubule stability (RASSF1A).

To date, chromosomal gains (+7) and losses (−10), allelic losses (10q, 9p), oncogene amplifications (MDM2, CDK4, EGF), and homozygous deletions and mutations of tumor suppressor genes (CDKN2A, TP53, PTEN, RB1, DMBT1) have been the most frequently found alterations in astrocytoma. However, methylation has been studied in a much lesser extent in astrocytic tumors. Recently, MGMT promoter hypermethylation has been associated to secondary glioblastoma phenotype and to an increase in GC→AT mutations in TP53 gene [6]. But promoter hypermethylation of other putative tumor suppressor genes has been less studied in astrocytomas, where LOH and mutational analysis have been prevalent.

PTEN, in 10q23.3, which has been demonstrated as a critical tumor suppressor gene frequently mutated in high-grade astrocytomas, has also been reported to be inactivated by methylation-dependent mechanisms in NSCLC [7] and carcinomas of the endometrium [8] and prostate [9]. Mutations on its coding region have been described in 30% high-grade astrocytomas, mainly in primary glioblastomas [10]. However, this mutation rate does not correlate with the higher frequency of LOH at 10q, which reaches 80–90% in both primary and secondary glioblastomas [11, 12]. Some authors then propose that (a) PTEN might be inactivated in high-grade astrocytomas by different mechanisms to LOH and mutations (e.g., promoter hypermethylation) and (b) other tumor suppressor genes might map 10q, near the PTEN locus.

Two important tumor suppressor *loci* map 9p21: CDKN2B (p15^INK4B^) and CDKN2A, which code for two alternative protein products, p14^ARF^ and p16^INK4A^. These three protein products negatively regulate cell cycle progression: p15 and p16^INK4A^ act in the CDK/cyclin/Rb pathway, while p14^ARF^ participates in the p14/MDM2/p53/p21 route. So, homozygous deletion of CDKN2A locus alters cell cycle regulation in two different but synergistic variants: p53 and Rb pathways. CDKN2A has a high frequency of homozygous deletions in high-grade astrocytomas, while mutations and promoter methylation of p14^ARF^ or p16^INK4A^ have a lesser importance in these tumors [13, 14].

Finally, recent reports have pointed at the RASSF1A gene, on 3p21.3 [15], as an important tumor suppressor gene inactivated in several neoplasms, such as NSCLC, melanoma, breast, kidney, bladder, and prostate cancer and in some pediatric tumors, like medulloblastoma, neuroblastoma, and Wilms' tumor [5]. RASSF1A is a Ras effector, whose function was thought to mediate the Ras apoptotic response; however, recent reports have demonstrated how RASSF1A is involved in maintaining cytoskeletal integrity [16] and regulating mitosis [17]. While mutations and homozygous deletions of its coding region are rare, promoter hypermethylation is the main cause of RASSF1A inactivation in cancer [15]. The possibility that alterations of RASSF1A may participate in the etiology of astrocytic tumors has not been much studied to date.

Our aim was to study the promoter methylation status of these five important tumor suppressor genes (p14^ARF^, p16^INK4A^, MGMT, PTEN, and RASSF1A), in a subset of astrocytomas and cell lines. While the methylation frequency of some of them (p14^ARF^, p16^INK4A^, and MGMT) has been widely assessed in high-grade astrocytomas, it has not been much studied in low-grade astrocytic tumors. Moreover, promoter hypermethylation as an inactivation mechanism of PTEN and RASSF1A genes has rarely been studied in astrocytomas.

## 2. Material and Methods

### 2.1. Tumor Specimens

A total number of 61 astrocytic tumors were analyzed by MSP; 23 were obtained from the General Hospital of the Medical University of Tianjin, Tianjin, China: 8 GBM, 6 AIII, and 9 AII; 26 were obtained from the Hospital de Navarra, Pamplona, Spain: 19 adult GBM, 2 AII, 4 AI, and 1 pediatric GBM. All these tumors had been frozen in liquid nitrogen after surgical extraction. Finally, 12 cases of high-grade pediatric astrocytomas included in paraffin were obtained from the Hospital Miguel Servet, Zaragoza, Spain.

### 2.2. Cell Lines

Astrocytoma cell lines U87 MG, T98G (grade IV), and MOG-G-CCM (grade III) were purchased from the European Collection of Cell Cultures (ECACC, Salisbury, UK); cell lines U251, U118 (grade IV astrocytomas), SW1783, and SW1088 (grade III astrocytomas) were purchased from the American Type Culture Collection (ATCC, Manassas, VA); finally, cell lines LN-405 (grade IV astrocytoma) and GOS3 (grade II/III astrocytoma/oligodendroglioma) were obtained from the Deustche Sammlung von Mikroorganismen und Zellkulturen GmbH (DSMZ, Braunchsweig, Germany). Cell lines were grown in RPMI supplemented with 10% FBS, 1% penicillin/streptomycin, and 5% MEM nonessential aminoacids. Medium was restored three times a week, and subcultures were done before cells reached confluence.

### 2.3. Nucleic Acids Extraction

DNA was obtained with phenol-chloroform purification from the frozen tissues. Paraffin embedded samples were taken into a series of alcohol in decreasing grade and, then, left until they were totally dried. Tissues were resuspended in 1 mL lysis solution buffer (0.5 M NaCl; 50 mM Tris-HCl, pH 7.6; 50 mM EDTA; 0.5% SDS) and were incubated with 20 *μ*L proteinase K (20 mg/mL) at 56°C, overnight. After that, 0.4 mL saturated NaCl were added to the mixture, shaked, and spun at 13.000 rpm, 10 min. Supernatant was carried to a new tube, and DNA was precipitated by adding 2 volumes of absolute ethanol at −80°C. The DNA was washed twice in 70% ethanol, dried, and resuspended in 200–500 *μ*L Tris-EDTA Buffer (10 mM Tris-HCl, 1 mM EDTA, pH 7.6).

RNA from cell lines was extracted with TRIzol Reagent (Invitrogen Ltd., Paisley, UK), following manufacturers' instructions.

### 2.4. Sodium Bisulfite Modification

Sodium bisulfite DNA modification was done as previously described [18]. One *μ*g DNA was mixed with 10 *μ*g Herry Sperm's DNA and 1,7 *μ*L 6 M NaOH to a final volume of 50 *μ*L and denatured at 37–42°C, 15 min. Then, 30 *μ*L 100 mM hydroquinone and 520 *μ*L 3 M sodium bisulfite pH 5.0 were added, and the reactions were incubated at 56°C for 16 h. To avoid liquid evaporation, eight drops of mineral oil were added to the mixture. Modified DNAs were recovered and purified by using the Wizard DNA Clean-Up System (Promega, Madison, WI), according to the manufacturer and eluted in 30–50 *μ*L Tris-EDTA Buffer (10 mM Tris-HCl, 1 mM EDTA, pH 7.6). These elutions were stored at −20°C until their use.

### 2.5. MSP

1.5–2.5 *μ*L modified DNA were used as template for each methylation-specific PCR. DNAs were added to mixes which contained 0.8 mM dNTPs, 2-3 mM MgCl_2_, 2.5 *μ*L 10X reaction buffer, 5–15 pmol primers, 5%  *μ*L DMSO, and 1 U AmpliTaq Gold DNA polymerase (Applied Biosystems, Foster City, CA). For p14^ARF^ and p16^INK4A^, amplification BioTaq DNA polymerase (Bioline Ltd., London, UK) was used, in a final volume reaction of 50 *μ*L. Five *μ*L 0.1 M *β*-mercaptoethanol were added to the PCR mix for p14^ARF^ amplification. Primers for MGMT and RASSF1A amplification were designed by using MethPrimer software [19] and are listed in Table 1. Primers for PTEN, p14^ARF^, and p16^INK4A^ (Table 1) had been previously described [8, 18].

### 2.6. RT-PCR

Five *μ*g RNA from each cell line were mixed with 2 *μ*L dNTPs, 20 mM, and 2 *μ*L random primers 250 *μ*M in a final volume of 10 *μ*L. The mixture was heated at 65°C, 10 min, and then 4 *μ*L 5X Reaction Buffer, 2 *μ*L 0.1 mM DTT, and 1 U RNAse Out (Invitrogen Life Technologies, Carlsbad, CA) were added. The mixture was heated at 42°C for 2 min and incubated with 1 U Superscript II RNA Retrotranscriptase (Invitrogen Life Technologies, Carlsbad, CA). Samples were heated at 42°C for 50 min, and a final step at 72°C for 10 min was used. cDNAs were diluted 1 : 5 and stored at −20°C until their use.

RT-PCR conditions for studying PTEN expression had been previously described [20]. For p14^ARF^, p16^INK4A^, MGMT, and RASSF1A expression, 1.5–2.0 *μ*L 1 : 5 diluted cDNA were used as template for each RT-PCR. cDNAs were added to a mixture that contained 0.8 mM dNTPs, 2.5 *μ*L 10X Reaction Buffer, 1.5 mM MgCl_2_, 5 pmol primers, and 1 U AmpliTaq Gold DNA polymerase (Applied Biosystems, Foster City, CA), in a final volume of 25 *μ*L. Primers were designed with Oligo 4.0 software (NBI, Hamel, MN) and are depicted in Table 1. An RT-PCR of the transferrin receptor gene (TFR) was done as internal control of the RNA amount.

## 3. Results

### 3.1. Adult Astrocytomas

#### 3.1.1. p14^ARF^ and p16^INK4A^

**Figure 1:**
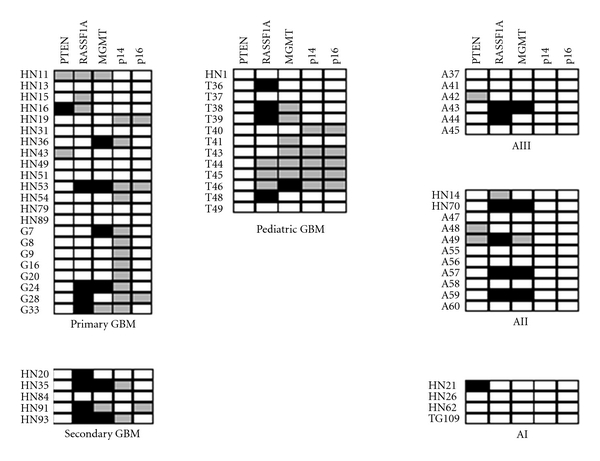
Methylation of PTEN, MGMT, RASSF1A, p16^INK4A^, and p14^ARF^ in adult and pediatric astrocytomas. Black boxes: presence of methylation; white boxes: absence of methylation; gray boxes: notdetermined. GBM: glioblastoma multiforme; AIII: anaplastic astrocytoma; AII: low-grade diffuse astrocytoma; AI: pilocytic astrocytoma.

Just 34 and 44 of the 48 adult astrocytomas could be assessed for p14^ARF^ and p16^INK4A^ promoter hypermethylation, respectively. We could not find any evidence of methylation in all samples tested (Figure 1).

p14^ARF^ hypermethylation could be studied in four astrocytoma cell lines (T98G, LN405, SW1783, and MOG-G-CCM), without finding any case of methylation (Table 2). p16^INK4A^ was studied in three cell lines (LN405, SW1783, and GOS3) without detecting any alteration. Cell lines U87MG, U118 MG, SW1088, and U251 MG did not show any interpretable band either for p14^ARF^ or for p16^INK4A^ methylation-specific PCR.

#### 3.1.2. PTEN

PTEN promoter status was analyzed in a total number of 43 adult astrocytomas (Figure 1). We could only find two cases of methylation: a primary glioblastoma (HN16), which represented 4% of all glioblastomas tested, and 1 pilocytic astrocytoma (HN21), which showed a slight signal of methylation.

**Figure 2:**
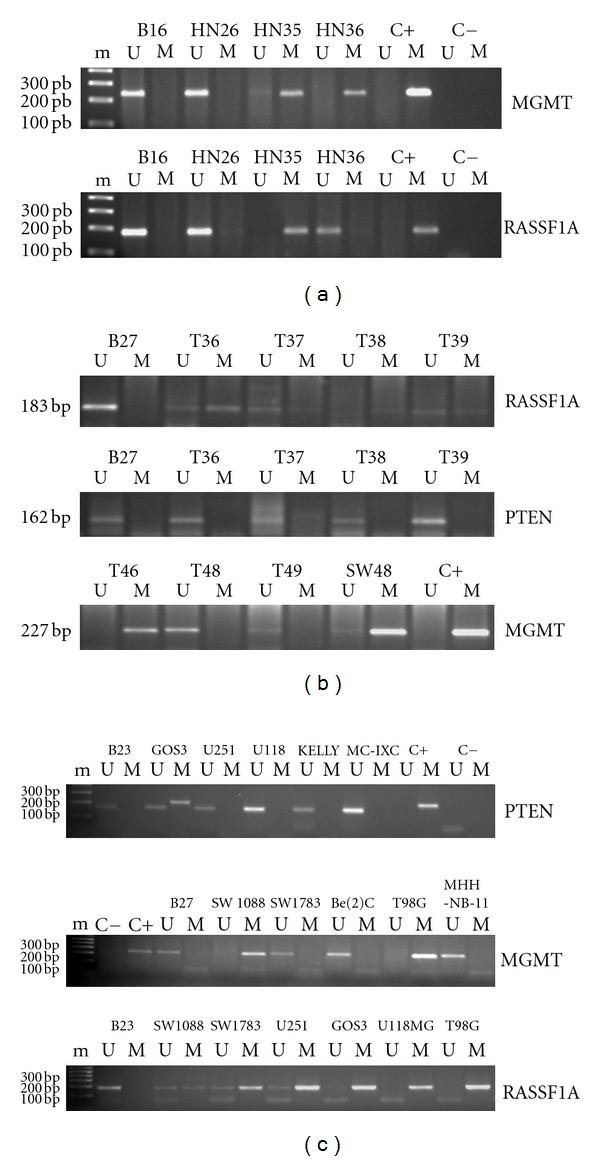
(a) MGMT and RASSF1A promoter hypermethylation in astrocytomas determined by MSP. PCR products were run in a 2% agarose gel stained with 0.5 *μ*g/mL ethidium bromide. B16: DNA obtained from blood of a normal donor; HN26: pilocytic astrocytoma; HN35: secondary glioblastoma; HN36: primary glioblastoma. m: 1 Kb plus DNA marker; U: unmethylated; M: methylated. C+: DNA methylated with Sss I (CpGenome, Intergen, Edimburgh, UK), positive control for methylation; C−: water. (b) RASSF1A, PTEN, and MGMT promoter hypermethylation in pediatric high-grade astrocytomas determined by MSP. PCR products were run in a 2% agarose gel stained with 0.5 *μ*g/mL ethidium bromide. B27: DNA obtained from blood of a normal donor; T36–T49: DNA from paraffin-embedded pediatric astrocytomas; SW48: colorectal cancer cell line with MGMT promoter hypermethylation, used as a positive control for DNA methylation; C+: Sss I methylated DNA (CpGenome, Intergen, Edimburgh, UK), used as a positive control for methylation; U: unmethylated; M: methylated. (c) PTEN, MGMT, and RASSF1A promoter hypermethylation in astrocytoma cell lines determined by MSP. PCR products were visualized in a 2% agarose gel stained with 0.5 *μ*g/mL ethidium bromide. B23: DNA from blood of a normal donor; GOS3: astrocytoma/oligodendroglioma cell line (grades II/III); U251: glioblastoma multiforme; U118: glioblastoma multiforme; SW1088: anaplastic astrocytoma; SW1783: anaplastic astrocytoma; T98G: glioblastoma multiforme; C+: DNA methylated with Sss I (CpGenome, Intergen, Edimburgh, UK), positive control for methylation; C−: water. Neuroblastoma cell lines Kelly, MC-IXC, Be(2)C, and MHH-NB-11 were included in the study. m: molecular weight DNA marker, 1 Kb Plus DNA ladder (Invitrogen, Life and Technologies, Carlsbad, CA); U: unmethylated; M: methylated. Some other cell lines, different to astrocytoma, appear in the figure.

We could study all cell lines and found two cases with methylation in one PTEN allele (cell lines GOS3 and MOG-G-CCM) (Figure 2(c), Table 2).

#### 3.1.3. MGMT

MGMT could be studied in 48 astrocytoma cases, and alterations were found in all grades but in pilocytic astrocytomas (Figure 1). Six glioblastomas (24%), 3 diffuse astrocytomas (30%), and 1 anaplastic astrocytoma (17%) showed MGMT promoter hypermethylation (Figure 2(a)). This gene also showed promoter hypermethylation in 7 of 8 astrocytoma cell lines analyzed (Figure 2(b), Table 2). U87MG cells could not be evaluated.

#### 3.1.4. RASSF1A

**Figure 3:**
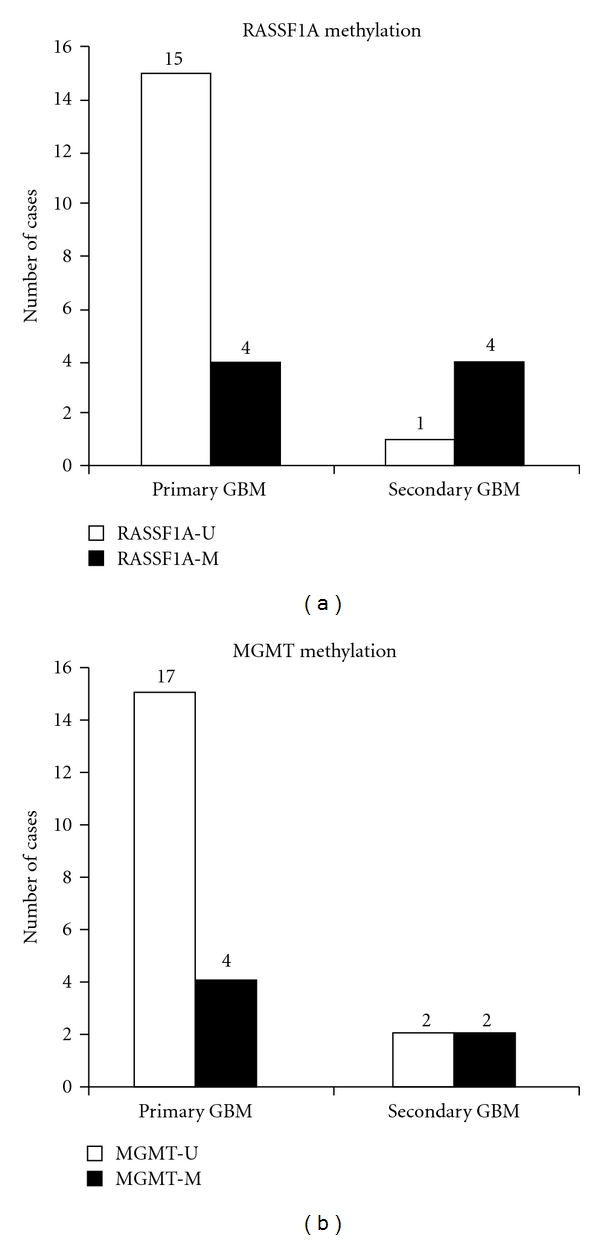
RASSF1A (a) and MGMT (b) methylation frequencies in primary and secondary adult glioblastomas. Horizontal axis: primary and secondary glioblastoma; vertical axis: number of cases studied. Black boxes: methylated cases; white boxes: unmethylated cases.

RASSF1A could be analyzed in 44 adult astrocytic tumors. This gene showed the highest frequency of hypermethylation in all grades studied, with the exception of pilocytic astrocytomas (Figure 1). Eight glioblastomas (33%), 2 anaplastic astrocytomas (33%), and 4 low-grade diffuse astrocytomas (40%) were hypermethylated for this gene (Figure 2(a)). We saw an statistically significant association between secondary glioblastoma phenotype and RASSF1A promoter methylation, as 4 of 5 tumors studied showed methylation of this gene; however, in the primary glioblastoma group, we could just detect 4 cases of methylation in 19 cases analyzed (Fisher's exact test, *P* = 0,028) (Figure 3). All cell lines showed RASSF1A methylation (Table 2).

### 3.2. Pediatric Astrocytomas

Thirteen high-grade pediatric astrocytomas were studied for methylation in the five genes (Figure 1). RASSF1A was the most frequently altered gene in this group, with 4 of 9 cases analyzed showing methylation (44%) (Figure 2(b)). MGMT showed promoter hypermethylation in 1 case of 6 studied (17%), while neither PTEN, p14^ARF^, or p16^INK4A^ showed methylation in any sample.

### 3.3. RT-PCR

We studied expression of both five genes in all astrocytoma and neuroblastoma cell lines by an RT-PCR approach (Table 2). Expression could not be evaluated in U118MG and U251 cells, as RNA was not available.

#### 3.3.1. RASSF1A

**Figure 4:**
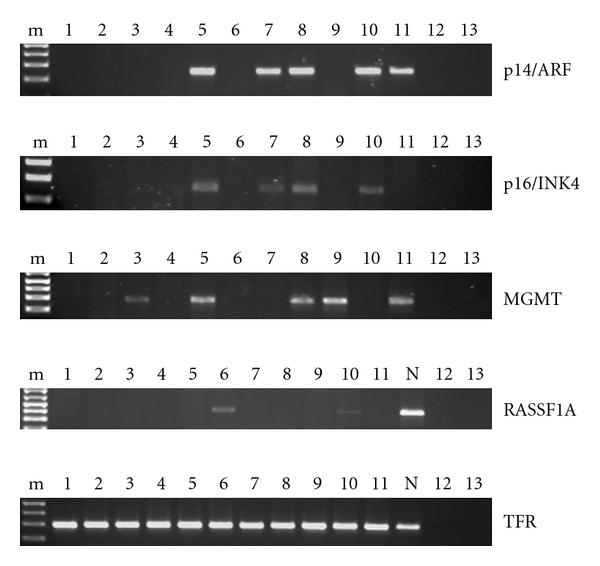
Expression of p14^ARF^, p16^INK4A^, MGMT, and RASSF1A in astrocytoma cell lines determined by RT-PCR. PCR products were visualized in a 2% agarose gel stained with 0.5 *μ*g/mL ethidium bromide. m: molecular weight marker: 1 Kb Plus DNA ladder; 1: U87MG; 2: A172; 3: H4; 4: GOS3; 5: SW1783; 6: SW1088; 7: CCF-STTG1; 8: MOG-G-CCM; 9: T98G; 10: LN 405; 11: SK-N-Be(2) (neuroblastoma); N: peripheral blood lymphocytes; 12: genomic DNA; 13: water. Cell lines A172 and H4 were not subjected to the methylation study. A fragment of the transferrin receptor gene (TFR) was reverse-transcribed as an internal control.

RASSF1A was the most frequently altered gene and lacked expression in all cell lines (Figure 4).

#### 3.3.2. PTEN

Astrocytoma cell line SW1088 lacked PTEN expression (not shown); however, this lack was not due to promoter hypermethylation, but to a homozygous deletion encompassing this gene (data not shown). Cell lines GOS3 and MOG-G-CCM, both presenting methylation in one PTEN allele, did not show a significant loss of expression when compared with other cell lines and a control with normal human astrocytes (NHA, Cambrex, East Rutherford, NJ).

#### 3.3.3. MGMT

Cell lines LN405, SW1088, and U87MG, which suffered MGMT promoter methylation, also showed lack of expression of this gene; on the contrary, SW1783 cells did not have MGMT hypermethylation and expressed the gene (Figure 4). We did not find correlation between MGMT methylation and lack of expression in cell lines MOG-G-CCM and T98G: both were methylated and expressed the gene.

#### 3.3.4. p14^ARF^ and p16^INK4A^

LN405, MOG-G-CCM, and SW1783 astrocytoma cells expressed p14^ARF^, while T98G, U87MG, GOS3, and SW1088 did not (Figure 4). A methylation-specific PCR approach could just be done for T98G, MOG-G-CCM, LN405, and SW1783, without finding any case of methylation. So, lack of expression in T98G cells seems not to be methylation-related.

In the case of p16^INK4A^ expression, just LN405, MOG-G-CCM, and SW1783 cells showed expression, while T98G, GOS3, SW1088, and U87MG did not (Figure 4). For this gene, only LN405, SW1783, and GOS3 could be evaluated by an MSP approach, without finding any evidence for methylation.

## 4. Discussion

In spite of the pivotal role of aberrant methylation described in other tumors [1], few are the studies which analyze promoter methylation of tumor suppressor genes in astrocytoma. However, methylation of some genes, like MGMT, has demonstrated to be critical, not only in tumorigenesis, but also in astrocytoma drug resistance [6, 21]. A recent report has found an increase of promoter hypermethylation in CALCA and CDH1 genes in high-grade astrocytomas, but they did not see any remarkable methylation frequency of other classical tumor suppressor genes, such as p14^ARF^, RB1, CDKN2B, or APC [22].

Baeza et al. [23] reported a methylation frequency of 35% for PTEN in high-grade astrocytomas and in 4 of 11 astrocytoma cell lines. They suggested that PTEN could also be inactivated by aberrant promoter methylation in glioblastomas, giving an explanation for the relatively low frequency of PTEN mutations discovered to date in these tumors. In our hands, however, PTEN promoter was not altered in the majority of glioblastomas analyzed, as only one of 25 cases studied presented a slight signal of methylation (4%). We could not find PTEN methylation either in high-grade or in low-grade astrocytomas, except one case of pilocytic astrocytoma, which showed a faint methylation signal. We consider this discrepancy unlikely to be due to different sample sizes, as we analyzed a total amount of 43 adult astrocytic tumors (25 GBM, 5 AIII, 9 AII, and 4 AI). It cannot be due to the fact that we analyzed another sequence in the CpG island of PTEN, as they also used two sets of primers including ours and obtained the same results. However, we could not reproduce our results when using their primers set (data not shown). Probably these differences are due to racial disparities or to the different geographic origin of individuals in both studies.

We did not find any case with p14^ARF^ or p16^INK4A^ promoter hypermethylation, either in high- and low-grade tumors or in pediatric astrocytic tumors. Homozygous deletion has been described as the major inactivating mechanism for the CDKN2A products [14], while several authors have found promoter methylation of p14^ARF^ and/or p16^INK4A^ in a subset of astrocytic tumors [24]. However, this frequency is quite low as compared with homozygous deletions. Several publications fail to find or just find a very low rate of methylation of these genes in astrocytomas [13, 25].

To date, the only gene for which methylation of its promoter has been considered quite important in astrocytoma tumorigenesis is MGMT. Nakamura et al. [6] reported an association between MGMT methylation-related silencing and an increase of GC→AT mutations in the TP53 gene in high-grade astrocytomas. They also found an association between MGMT hypermethylation and secondary glioblastoma phenotype. In spite of the fact that we do not find a significant association, our results are rather similar to those of Nakamura et al., as we found MGMT promoter hypermethylation in 15% primary glioblastomas, 50% secondary glioblastomas (2 of 4 samples analyzed), and 33% low-grade diffuse astrocytomas. In this case, the discrepancy with Nakamura et al. may be due to the different sample size (we analyzed a total number of 40 adult astrocytomas, while 106 astrocytomas were studied by Nakamura et al.). We could also detect one case of pediatric high-grade astrocytoma with MGMT hypermethylation, representing 17% of cases analyzed (6 cases).

RASSF1A showed the highest methylation frequency in all grades of astrocytoma studied (33% of high-grade (8 GBM and 2 AIII) and 40% of low-grade adult astrocytomas were methylated). Moreover, RASSF1A hypermethylation significantly associated to secondary glioblastoma phenotype, as 4 of 5, secondary glioblastomas, suffered hypermethylation on its promoter, while only 4 of 19 primary glioblastomas did so (*P* = 0.028, Fisher's exact test). Recently, RASSF1A promoter methylation has been revealed as a frequent epigenetic alteration in high-grade astrocytomas [26, 27]. However, those authors do not specify any clinical data of their tumors, so they cannot confirm whether methylation of this gene is associated to primary or secondary glioblastoma.

RASSF1A was also the most frequently altered gene in pediatric high-grade astrocytomas, as 4 of 9 cases presented methylation of this gene; however, not all pediatric samples could be evaluated for methylation in the five genes, as DNA extracted from archival material is of worse quality than DNA from frozen samples.

Recent reports have described frequent RASSF1A hypermethylation in pediatric tumors as medulloblastoma, neuroblastoma, and Wilms' tumor [5]. Wong et al. [28] proposed that RASSF1A promoter hypermethylation could be one of the first steps in pediatric tumorigenesis. However, it is still unclear whether RASSF1A inactivation is an early step in the etiology of embryonic tumors, or whether methylation of its promoter is a secondary effect of a disregulated cell proliferation, which can confer selective advantage to the cancer cell. Recently, this gene has been found methylated in nontumorigenic tissues from normal population, in association to aging [29]. In this way, Li et al. [30] found an association between N33 and ER methylation in older glioblastoma patients, but not in younger patients; both these genes are more often methylated in normal older individuals than in younger ones. In spite of this, authors generally agree on the selective advantage that RASSF1A inactivation confers to cancer cells, as well as on growth suppression in breast, prostate, and lung cancer cells through RASSF1A expression [31, 32].

## 5. Methylation and Expression in Cell Lines

We could detect methylation in cell lines in much higher frequency than in primary tumors; however, the methylation profile of the cell lines correlated with the profile in primary tumors. A higher frequency of tumor suppressor gene promoter hypermethylation in cell lines than in primary tumors has also been reported previously [33]; this can be due to the selective growth advantage that methylation-related silencing of tumor-suppressor genes confers to cancer cells, which can favor clonal selection of cells with aberrant methylation patterns in successive subcultures.

In our hands, only GOS3 and MOG-G-CCM cell lines were hypermethylated for PTEN. However, Baeza et al. [23], who also studied PTEN methylation in astrocytoma cell lines, found methylation of this gene both in U87 MG and T98G cells, which were free of methylation in our study. They used the same set of primers than us, but maybe a higher number of passages in their cell lines that could have promoted PTEN methylation due to successive cell replications. Like us, they did not find PTEN hypermethylation in U251 cells.

RASSF1A was the only gene for which we could see a good correlation between promoter hypermethylation and reduction or lack of expression. All astrocytoma cell lines were methylated for RASSF1A, and none of them expressed the gene (only SW1088 and LN405 cells showed a faint signal of expression). On the contrary, in the case of PTEN, we could not see a reduction of mRNA expression in GOS3 and MOG-G-CCM cells, which had previously shown to be hypermethylated for this gene; however, they both had just one methylated allele, the other one remaining free of methylation, which might be enough to maintain normal PTEN expression levels.

SW1783, MOG-G-CCM, and LN405 cells expressed the p14^ARF^ and p16^INK4A^ genes, while U87MG, GOS3, SW1088, and T98G did not. All cell lines that expressed these genes did not show any detectable methylation signal. U87MG and SW1088 cells did not produce a band for p14^ARF^ and p16^INK4A^ MSP; this might be due to a big homozygous deletion that affected their promoter. Other mechanisms different from methylation, like homozygous deletion, can also be responsible of p14^ARF^ silencing in U87 MG and SW1088 cells [34]; however, this possibility has not been studied. We cannot find an explanation for the expression of p16^INK4A^ in MOG-G-CCM cells, when the MSP approach did not give any interpretable result: maybe MSP could not work as well as in other experiments, or a mutation in the priming site, that did not affect gene expression, was the cause of data misinterpretation after MSP.

In the case of MGMT, although 7 cell lines showed methylated or hemimethylated alleles, only three of them lacked expression, while MOG-G-CCM and T98G retained the MGMT transcript (expression was not evaluated in U118 and U251 cell lines).

## 6. Conclusion

Our results suggest the existence of mechanisms of inactivation different from methylation for p14^ARF^ and p16^INK4A^ genes, while offer doubts about the direct cause-effect relationship between promoter methylation and lack of expression of PTEN and MGMT in astrocytoma cell lines. This lack of correlation between PTEN hypermethylation and lack of expression has partially been described by Baeza et al. [23] by immunohistochemistry in primary human astrocytomas. However, RASSF1A might be a main target of methylation-related inactivation in human astrocytoma. Methylation of RASSF1A might be a diagnosis marker for secondary glioblastomas and for pediatric astrocytomas. Being a Ras effector that mediates Ras apoptotic response, RASSF1A methylation can be an alternative mechanism of the Ras/MAPK/ERC pathway alteration in those tumors with intact Ras.

## Figures and Tables

**Table 1:** Primers and conditions for PCRs.

Gene	Primer sense	Primer antisense	Size	T (°C)	Cycles
PTEN					
-Methylation					
-U	5′-GTGTTGGTGGAGGTAGTTGTTT-3′	5′-ACCACTTAACTCTAAACCACAACCA-3′	162 bp	62°C	38
-M	5′-TTCGTTCGTCGTCGTCGTATTT-3′	5′-GCCGCTTAACTCTAAACCGCAACCG-3′	206 bp	62°C	38
MGMT					
-Methylation					
-U	5′-GAGAGATTTGTGTTTTGGGTTTAGTG-3′	5′-CCTTCAACCAATACAAACCAAACAA-3′	236 bp	62°C	38
-M	5′-ATTCGCGTTTCGGGTTTAGC-3′	5′-CGACCGATACAAACCGAACG-3′	227 bp	62°C	38
-Expression	5′-GGGGAAGCTGGAGCTGTCTG-3′	5′-TCTCCGAATTTCACAACCTTCA-3′	282 bp	62°C	32
RASSF1A					
-Methylation					
-U	5′-GAGAGTGTGTTTAGTTTTGTTTTTG-3′	5′-CCCATACTTCACTAACTTTAAACAC-3′	183 bp	56°C	38
-M	5′-GAGAGCGCGTTTAGTTTCGTTTTC-3′	5′-ACCCGTACTTCGCTAACTTTAAACG-3′	184 bp	62°C	35
-Expression	5′-TCTGTGGCGACTTCATCTGG-3′	5′-TTGGGCAGGTAAAAGGAAGT-3′	424 bp	60°C	35
p14^ARF^					
-Methylation					
-U	5′-TTTTTGGTGTTAAAGGGTGGTGTAGT-3′	5′-CACAAAAACCCTCACTCACAACAA-3′	132 bp	62°C	35
-M	5′-GTGTTAAAGGGCGGCGTAGC-3′	5′-AAAACCCTCACTCGCGACGA-3′	122 bp	58°C	35
-Expression	5′-CCGCCGCGAGTGAGGGTTTT-3′	5′-GCACGGGTCGGGTGAGAGTGG-3′	242 bp	65°C	32
P16^INK4A^					
-Methylation					
-U	5′-TTATTAGAGGGTGGGGTGGATTGT-3′	5′-CAACCCCAAACCACAACCATAA-3′	150 bp	62°C	35
-M	5′-TTATTAGAGGGTGGGGCGGATCGC-3′	5′-GACCCCGAACCGCGACCGTAA-3′	151 bp	65°C	35
-Expression	5′-CGCGCGTACAGATCTCTCGAA-3′	5′-CACGGGTCGGGTGAGAGTGG-3′	161 bp	68°C	35
TFR					
-Expression	5′-GTCAATGTCCCAAACGTCACCAGA-3′	5′-ATTTCGGGAATGCTGAGAAAACAGACAGA-3′	298 bp	60°C	30

U: unmethylated; M: methylated.

**Table 2:** Promoter hypermethylation and expression in cell lines.

Cell line	Diagnosis	Methylation					Expression				
		PTEN	MGMT	RASSF1A	p14	p16	PTEN	MGMT	RASSF1A	p14	p16
T98G	GBM	−	■	■	−	*	+	+	−	−	−
LN-405	GBM	−	■	□	−	−	+	−	−/+	+	+
U118	GBM	−	■	■	*	*	§	§	§	§	§
U251	GBM	−	■	■	*	*	§	§	§	§	§
U87	GBM	−	■	■	*	*	+	−	−	−	−
MOG-G-CCM	AIII	□	■	■	−	*	+	+	−	+	+
SW1088	AIII	−	■	□	*	*	−	−	−/+	−	−
SW1783	AIII	−	−	■	−	−	+	+	−	+	+
GOS3	A/O (II/III)	□	−	■	*	−	+	−	−	−	−

GBM: glioblastoma multiforme; AIII: anaplastic astrocytoma; A/O: astrocytoma/oligodendroglioma. ■: methylated; □: hemimethylated; +: expression; −/+: low expression; −: lack of expression/absence of methylation; *: nonanalyzed; §: nonstudied.

## Abbreviations

AI: Pilocytic Astrocytoma
AII: Low grade diffuse Astrocytoma
AIII: Anaplastic Astrocytoma
ARF: Alternative Reading Frame
CDKN2A: Cyclin-Dependent Kinase Inhibitor 2A
dNTPs: Deoxynucleotides Tris-Phosphate
EDTA: Ethylene-Diamine Tetraacetic Acid
GBM: Glioblastoma Multiforme
FBS: Fetal Bovine Serum
MGMT: O6-Methylguanine-methyltransferase
MSP: Methylation Specific PCR
MEM: Minimum Essential Medium
RASSF1A: Ras Association Family Protein 1A
RPMI: Roswel Park Memorial Institute media
RT-PCR: Reverse-Transcribed PCR
SDS: Sodium Dodecyl sulphate
PCR: Polymerase Chain Reaction
PTEN: Phosphatase and Tensin homolog located in chromosome ten
DMBT1: Deleted in Malignant Brain Tumors-1
LOH: Loss of Heterozygosity
NSCLC: Non-Small-Cell Lung Carcinoma
TFR: Transferrin Receptor gene.
